# The habitat preferences of invasive raccoon dog imply elevated risks for wetland-associated prey species

**DOI:** 10.1007/s00442-024-05614-y

**Published:** 2024-09-04

**Authors:** Pyry Toivonen, Toni Laaksonen, Antti Piironen, Vesa Selonen

**Affiliations:** Department of Biology, University of Turku, 20014 Turku, Finland

**Keywords:** Invasive species, Mesopredators, Conservation, Waterfowl, Habitat selection

## Abstract

Habitat preferences of invasive predators determine where and for which prey species they pose a threat upon. This is crucial information for the conservation of endangered prey species because invasive predators pose additional predation on top of that caused by natural predators. In large parts of Europe, the most common invasive mesopredator is the raccoon dog (*Nyctereutes procyonoides*). To understand the risk that the raccoon dog poses for wetland-associated species, we collected information about its habitat preferences near these habitats. We used data on 24 GPS-collared raccoon dogs from three landscape types in Finland, to study their spatial and temporal habitat preferences. We first determined their home ranges, within which we then examined habitat use and preferences. Raccoon dogs showed generalist habitat use, which was evident in their wide range of used habitats. However, in spring and summer, during the breeding seasons of waterfowl and amphibians, they preferred wetlands and peatlands. They also preferred shorelines and the edges of forests and agricultural fields. During autumn and winter, raccoon dogs did not prefer wetlands. These findings support the conclusion that the raccoon dog’s habitat preferences pose a particular threat to wetland-associated species, such as nesting waterfowl and amphibians. The species’ habitat preferences coupled with high numbers of this invasive mesopredator pose additional predation for endangered wetland-associated species on top of that of native predators.

**Supplementary Information:**

The online version contains supplementary material available at 10.1007/s00442-024-05614-y.

## Introduction

Predation is one of the most direct ways, through which invasive species affect native species (Doherty et al. 2016). Not all alien species are harmful, but invasive mammalian predators are concluded to be particularly harmful to many native species (Pitt and Witmer 2007; Hurd 2008; Doherty et al. 2016). Invasive predators may have even doubled the impact of native predators on vertebrate prey (Salo et al. 2007). For example, in Australia invasive mammalian predators, such as feral cats (*Felis catus*) and red foxes (*Vulpes vulpes*) cause serious harm to native marsupials (Doherty et al. 2017). Another group of endangered prey, for which increasing invasive predator populations appear to cause declines, are ground-nesting birds (Dahl and Åhlen 2018; Nummi et al. 2019; Koshev et al. 2020; McMahon et al. 2020; Pöysä and Linkola 2021; Jaatinen et al. 2022). The decline of ground-nesting birds appears to be a continent-wide phenomenon in Europe (McMahon et al. 2020).

One of the most common invasive mesopredators in Europe is the raccoon dog (*Nyctereutes procyonoides*). The raccoon dog spread to Europe from the former Soviet Union where it was introduced from Eastern Asia (Kauhala and Kowalczyk 2011; Kauhala and Saeki 2016). To Finland, they spread during the 1950s–1960s (Finnish Biodiversity Information Facility 2020), and northern Finland and Sweden are part of their current invasion front. In the EU, it is listed as a species of Union concern (European Commission 2020). Raccoon dogs are believed to be a threat to birds nesting in wetlands and shorelines, and their role as a nest predator has been the subject of recent interest (e.g. Nummi et al. 2019; Holopainen et al. 2020, 2021; Selonen et al. 2022a). However, studies on the raccoon dogs’ effect on the breeding or nesting success of ground-nesting birds have been contradictory (e.g. Kauhala 2004; Nummi et al. 2019; Jaatinen et al. 2022). The data indicating the negative impacts of raccoon dogs on birds is much based on artificial nest predation studies (Dahl and Åhlén 2018; Nummi et al. 2019; Holopainen et al. 2021). In addition to ground-nesting birds, amphibians may be negatively affected by raccoon dogs (Kauhala and Kowalczyk 2011). Indeed, a recent diet analysis observed frogs to be the common food of raccoon dogs in wetland areas in southern Finland (Tuomikoski et al. 2024).

The raccoon dog has an opportunistic diet, consisting of plant material, invertebrates, rodents, amphibians, fishes and birds (Mulder 2012). According to studies on stomach contents and morphological identification of prey remains in scats, birds eaten by raccoon dogs are mainly chicks. There has been debate on how much raccoon dogs eat bird eggs (e.g. Kauhala and Kowalczyk 2011; Dahl and Åhlén 2018). The egg material may not be seen in stomach content analyses and could thus be underrepresented (Dahl and Åhlén 2018, but see Tuomikoski et al. 2024). At the same time, raccoon dogs are common predators of artificial nests, suggesting that they could eat substantial numbers of bird eggs (Dahl and Åhlén 2018; Holopainen et al. 2021). Since the raccoon dog is an opportunistic predator, studying its habitat selection and use may provide us with more information on what kind of food sources they come upon and are therefore able to consume. The spatiotemporal preferences in habitat use may indicate an elevated risk of predation pressure for potential prey species, such as wetland-associated bird species and amphibians.

Generalist predators, such as the raccoon dog, have a versatile way of using habitats, which is influenced by the mere availability of habitats (Roever et al. 2012). Indeed, this ability is a major reason for why generalist species often become successful invaders (Hurd 2008). However, even generalist species exhibit some level of optimization in their habitat use, which leads to selection and specific risks for prey associated with these selected habitats. For raccoon dogs varying levels of habitat selection have been observed (e.g. Drygala et al. 2007; Kauhala and Auttila 2010; Sutor and Schwarz 2013). For example, in the hemiboreal and southern boreal zones, they have been observed to prefer mosaics of deciduous or mixed forests, damp meadows, fields and gardens (Kauhala and Auttila 2010, see also e.g. Drygala et al. 2007; Kauhala et al. 2007; Kauhala and Auttila 2010; Sutor and Schwarz 2013; Herfindal et al. 2016). The observed selection of habitats by an animal is, however, heavily influenced by the spatial (Manly et al. 2002) and temporal (Roever et al. 2012) scale of the study, and by the methodologies used to measure the use and availability of habitats.

We use data from GPS-tracked raccoon dogs to study the habitat preferences of the species during spring and summer, which is the breeding season of ground-nesting birds and amphibians. We use data from three study regions that represent different landscapes with different habitat compositions (northern, southern coastal and southern inland landscapes). We analyze whether the home range sizes differ between the three landscape types and whether the habitat preferences differ between seasons. If raccoon dogs are (as suspected) a particular risk for wetland-associated species, we presume that (i) they prefer wetlands, riverbanks and shorelines in their habitat use, and (ii) the preference for the aforementioned habitats is stronger during the breeding season than the non-breeding season of waterfowl and amphibians. We also expect raccoon dogs to prefer habitat edges, as observed for other mesopredators (Šálek et al. 2010; Bischof et al. 2018).

## Material and methods

### Study areas and landscapes

**Fig. 1:**
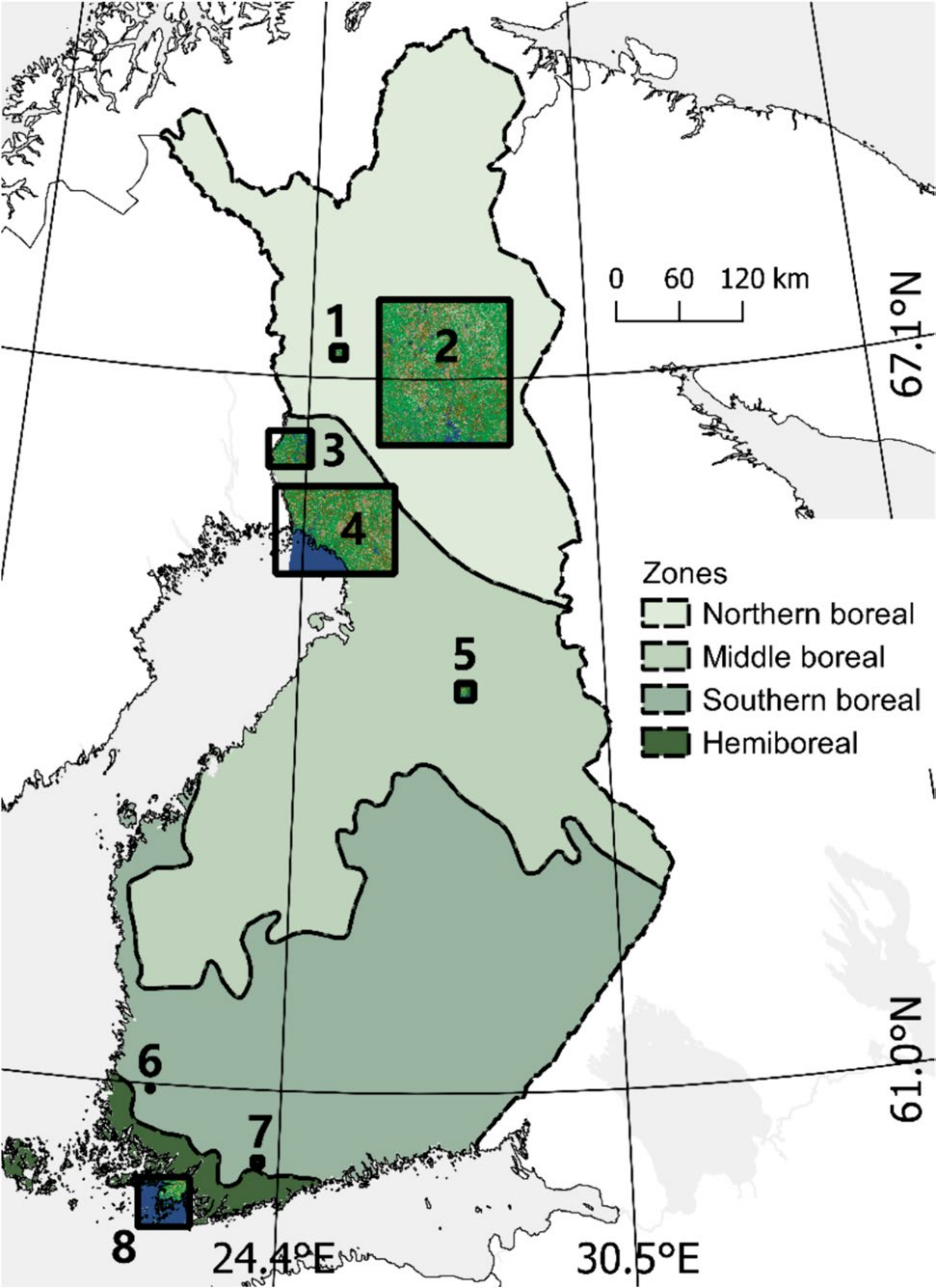
The eight study areas representing the three study landscapes. The northern region has five areas (1–5), the southern inland region has two areas (6–7) and the southern coastal region has one area (8). Areas vary in size depending on the number of raccoon dogs in the area and their home range size

We use data from eight different areas representing three different landscape regions (Fig. 1). The southern coastal region is represented by one area in the Baltic Sea coastal area in southwestern Finland around Kemiönsaari (hemiboreal zone, appr. 60.0° N, 22.5° W), the southern inland region is represented by two small areas in Lohja (appr. 60.39° N, 24.04° W) and Eura (appr. 60.96° N, 22.10° W) in inland regions of southern Finland (southern boreal zone), and the northern region is represented by five large areas in northern Finland (northern and middle boreal zones, centroid appr. 66.7° N, 26.4° W). These regions represent different landscapes, climates, population densities and phases of invasion. Raccoon dog densities are lower in the northern parts of the country (Helle and Kauhala 1991).

### GPS-tracking

Raccoon dogs were tracked with Global Positioning System (GPS) transmitters from two different manufacturers (Followit and Arctic-iot). Both collars (Followit Ultra Light GSM/GPRS and Arctic-iot Naltio) weighed less than 5% of the weight of the animal (211 g and 102 g, respectively). The data does not have any precise, uniform tracking periods (supplementary Table 1) and was influenced by the killing of raccoon dogs by hunters. That is, tracking often ended in the death of the individual and partner removal of some of the tracked individuals may have increased their movement activity. The purpose of the tracking of these individuals was to find out nesting sites, to remove their partners by wildlife managers (we take the partner removal into account in the home range analysis, see below). Most of the locations were recorded during nighttime. Many attempts to record locations during daytime were unsuccessful, probably due to poor GPS signal inside underground nests where the raccoon dogs spent their day. However, also daytime observations were made because raccoon dogs sometimes are also active during the day (Zoller and Drygala 2013). The mean location interval was 3.5 h in the southern inland region, 6.9 h in the southern coastal region and 8.4 h in the northern region. The data for the southern inland region came from a different project than the rest of the data and the difference between the southern coastal and northern regions is explained by the difference in successful location attempts (coastal region having more success).

To compare the habitat selection between seasons, we divided the tracking data into two periods. The spring/summer season included the locations from the breeding season of ground-nesting birds and amphibians (southern regions: April–July, northern region: May–July) and the autumn/winter season included the remaining locations (southern regions: August–March, northern region: August–April). The raccoon dogs in Finland do remain active also in winter, although they use periodically winter sleep during the coldest periods (Mustonen and Nieminen 2018; Selonen et al. 2024a, b). Unfortunately, the current data was insufficient to properly separate autumn and winter seasons in all the regions (see supplementary Figs. 3, 4 and 5).

We filtered the data by the number of satellites used to record each location, with four or more satellites (three-dimensional) being acceptable (Lewis et al. 2007). The estimated error given by the most inaccurate collars was around 20 m, which is partly covered by the resolution of the habitat rasters (16 × 16 m). Some of the tracked individuals were paired with each other, and because partners move together and use the landscape in a very similar manner, only the partner with the most fixes was used, to avoid pseudoreplication. Due to some migratory or nomadic movements, two individuals produced abnormally large home-range estimates, likely not representing a stationary home range. After removing these outliers from seasonal home-range data, we had in total 16 493 locations from 25 individuals (13 males, 12 females) from the years 2017–2022 that we used in the analyses (see supplementary Table 1). 3397 locations were distributed to the southern inland region, 6415 locations to the southern coastal region and 6681 locations to the northern region. 24 of these individuals were used to study the home ranges and habitat selection during the spring/summer season (8405 locations), 21 during the autumn/winter seasons (8088 locations) and 20 to compare between the seasons (15,132 locations).

### Home ranges

We estimated the home ranges of the raccoon dogs using autocorrelated kernel density estimation (AKDE, Fleming et al. 2015) that takes the autocorrelation between fixes into account. AKDE also takes tracking intervals, which differed between the regions, into account. Estimation was done on two utilization distributions (UD): 95% (general home range) and 50% core area of the home range), the latter being the estimation for core areas.

To analyze variation in home range sizes, we used linear models with home range size (95% UD) as the response variable. We first fitted a linear regression model with the following explanatory variables: number of GPS fixes, study region, proportion of GPS fixes inside water-associated habitats (WAH: peatlands, banks and shorelines and wetlands), and the interaction of the latter two. We only used data from the spring/summer season (breeding season of waterfowl and amphibians) for this model. With this model, we tested whether raccoon dogs that used more water-associated habitats would have smaller home ranges. In other words, we expected a negative correlation between the use of WAH and home range size. We found that neither the number of GPS fixes or the use of WAH influenced the home range sizes (*t* = 0.82, *P* = 0.43 and *t* = 0.11, *P* = 0.92, respectively, *n* = 24). We then tested the effect of partner removals on the size of the home range as partner removal can increase movement by activating partner-seeking behavior. For this purpose, we made a linear model where only the individuals who had data about partner removals were included (see supplementary Table 1). The southern inland region was excluded because no partner removals were executed there. We found that partner removals did not have a significant effect on the home range size either (*F*_1,13_ = 2.41, *P* = 0.14, *n* = 16). These variables were, therefore, not considered in the final model testing home range sizes.

Finally, for the effect of region and season on home range size, we fitted a repeated measures ANOVA with only the study region, season and their interaction as predictors and individual ID as the within-subject term.

To achieve a normality of residuals, we transformed the area to a logarithmic scale for all the fitted models. The effect of sex was not obvious and sex was omitted, as earlier studies have shown that raccoon dogs do not have sexually dimorphic differences in how they use their environment (Drygala et al. 2007; Saeki et al. 2007; Melis et al. 2015; Herfindal et al. 2016).

### Habitat classification

We created habitat rasters for each tracking year for the area within the home ranges of all raccoon dogs in each of the three study regions. Each tracking year had its own raster because the landscape in some areas changed between the years due to logging. We used the Topographic Database produced by the National Land Survey of Finland (NLS, downloaded 7/2022) to define the built area, roads, agricultural fields, lakes and sea, peatlands and rivers and streams. MS-NFI forest canopy cover (2017 and 2019) and forest land class datasets produced by Natural Resources Institute Finland (Luke) were used to define forests, open forest areas and open rock. Forest use declarations produced by the Forest Centre were used to detect changes in the forest cover between and after the years of 2017 and 2019. Land Cover on Wetlands 2017 dataset produced by the Finnish Environment Institute (SYKE) was used to define the wetland habitats. In addition to these datasets, satellite images produced by the ESA Sentinel-2 MSI satellite and orthophotos from 2021 produced by NLS and Corine Landcover 2018 data were used as supporting datasets to confirm habitat classifications, e.g. for manually confirming yearly logging events in forests. In northern Finland, no satellite data or orthophotos were available for all the years and areas, so open forest and logging areas were classified only by the canopy cover given by the MS-NFI 2017 and 2019 datasets. This was done in the coastal region as well in addition to confirming logging events because coastal landscapes have lots of naturally open forest areas which would not show in any other dataset. Finally, we defined two-pixel-wide edges (16 m straight to both sides of the edge) for agricultural fields and forest patches, and water bodies using a method described by Metzger and Muller (1996). For this method, we used a cross-shaped 3 × 3 focal window and produced the final versions of habitat rasters for each area and year. The final habitat data consists of 14 habitat classes (Table 1) with the resolution of 16 × 16 m. For comparison between seasons, we removed the habitat classes forest edge and open forest because of technical reasons limiting their use for seasonal comparison.

**Table 1:** Distribution of GPS fixes of raccoon dogs in different habitat classes and availability of different habitats in three study regions during the spring/summer season

Habitat	Southern inland region		Southern coastal region		Northern region	
	GPS fixes (%, SD)	Availability (%, SD)	GPS fixes (%, SD)	Availability (%, SD)	GPS fixes (%, SD)	Availability (%, SD)
Built area	6.7 (**± **4.5)	7.2 (**± **3.2)	4.0 (± 3.9)	2.0 (**± **1.9)	1.0 (**± **1.1)	1.3 (**± **1.2)
Roads	3.9 (**± **3.6)	5.3 (± 2.0)	2.4 (**± **2.8)	2.0 (**± **2.5)	2.0 (**± **1.7)	3.0 (**± **1.9)
Agricultural fields	7.9 (**± **7.8)	22.1 (**± **9.2)	3.9 (**± **3.5)	3.6 (**± **4.4)	7.8 (**± **11.1)	4.5 (**± **3.9)
Field edges	7.0 (± 5.2)	6.9 (**± **2.0)	4.9 (**± **4.0)	2.3 (**± **2.4)	9.5 (**± **8.1)	2.7 (**± **1.9)
Forests	28.9 (**± **19.1)	27.4 (**± **12.6)	19.5 (**± **4.7)	17.3 (**± **10.3)	21.3 (**± **12.9)	35.2 (**± **9.1)
Forest edges	8.5 (**± **5.6)	5.8 (**± **2.5)	25.0 (**± **7.5)	19.3 (**± **6.5)	19.8 (**± **6.9)	16.7 (± 2.0)
Open forest area	5.2 (**± **4.8)	5.2 (**± **1.9)	14.5 (**± **8.7)	8.7 (**± **7.4)	5.5 (**± **3.7)	5.4 (**± **1.7)
Lakes and sea	0.2 (**± **0.6)	1.7 (**± **2.7)	0.5 (**± **0.5)	27.9 (**± **26.2)	*NA*	*NA*
Banks and shoreline	1.8 (**± **2.3)	2.3 (**± **1.6)	12.0 (**± **8.8)	7.9 (**± **6.8)	4.4 (**± **5.7)	2.1 (± 3.0)
Wooded peatland	1.0 (**± **1.5)	4.1 (**± **6.1)	2.1 (**± **2.4)	0.7 (**± **0.6)	21.0 (**± **17.2)	17.2 (**± **9.1)
Treeless peatland	12.9 (**± **25.3)	2.6 (**± **3.6)	0.7 (**± **1.3)	0.3 (**± **0.4)	6.6 (**± **3.4)	8.2 (**± **6.6)
Rivers and streams	4.1 (**± **6.2)	1.9 (**± **1.5)	0.0	0.0	0.0 (**± **0.1)	1.9 (**± **3.0)
Wetlands	9.4 (**± **22.9)	7.6 (**± **10.1)	2.9 (**± **3.0)	1.6 (**± **1.2)	3.6 (**± **9.3)	1.2 (**± **3.2)
Open rock	0.0	0.0	7.7 (**± **9.4)	6.5 (**± **6.9)	0.0 (**± **0.1)	0.4 (**± **0.5)

Availability proportions are calculated inside the home ranges (95% utilization distribution) of individuals. GPS fixes = proportion of GPS fixes inside each habitat. Availability = proportion of each habitat calculated from randomized points

**Fig. 2:**
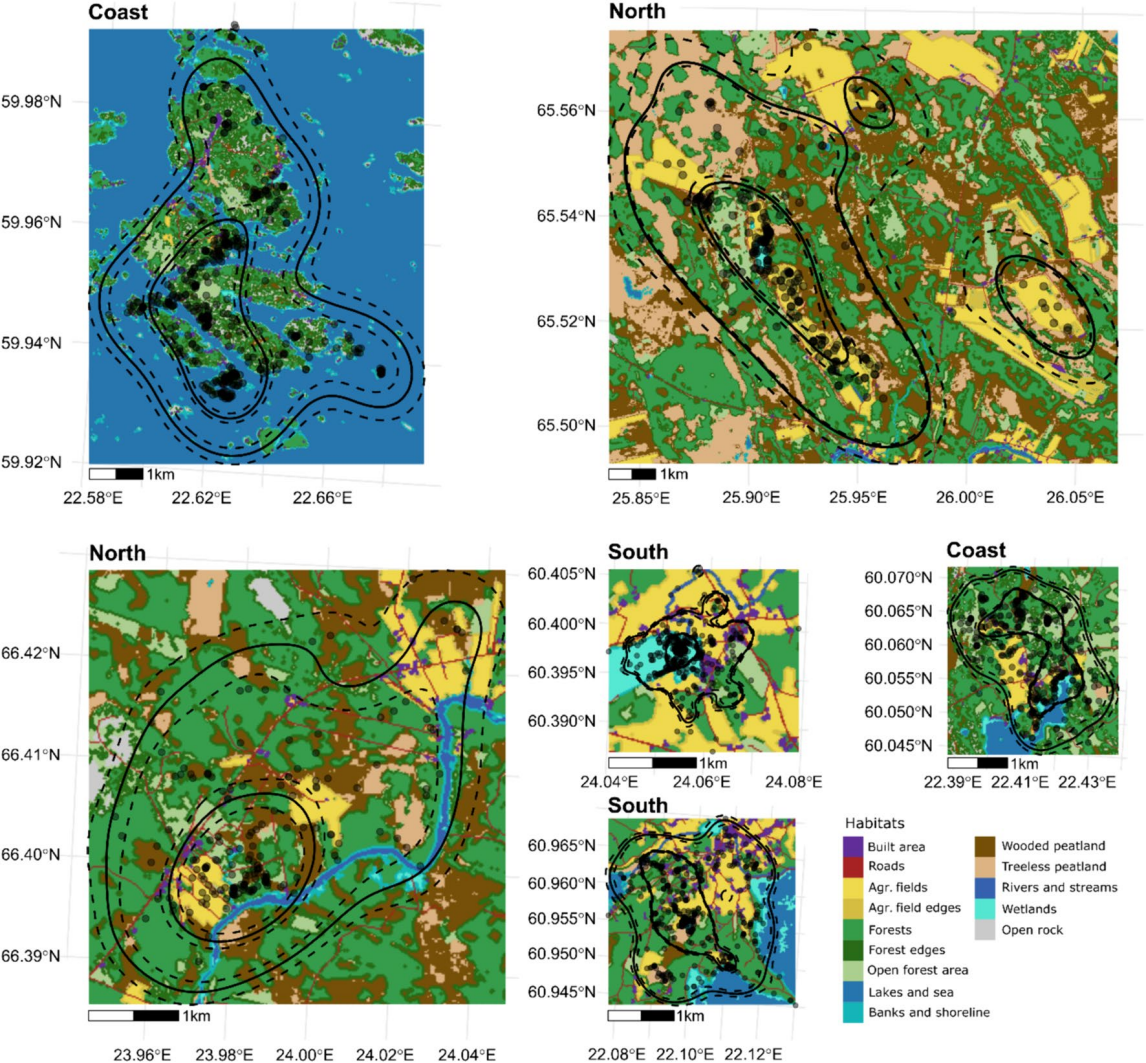
GPS locations and estimated home ranges for six individuals during the spring/summer season (1 April–31 July), two from each region (south, coast, north). The outer black line in each UD denote the estimated mean home range (95% UD), and the inner black line denotes the estimated mean core area (50% UD). The dashed lines represent the 95% CI for each estimate, and the dots denote the GPS locations for each individual. On the background are the habitat rasters for each individual. The legend denotes all the habitat classes and their representative colors

The habitat rasters were made using both QGIS (v. 3.16.16) and the raster package’s (v. 3.6-3, Hijmans 2022) merging and focal-window tools in R (R Core Team 2022). For examples of home ranges and habitat rasters see Fig. 2.

### Habitat selection

We used resource selection functions (RSFs) to compare the distribution of raccoon dog GPS fixes in relation to habitats available within the home range of each individual. The model parameters were estimated using infinitely weighted logistic regression as described by Fieberg et al. (2021). Results of logistic regression were interpreted and visualized with odds ratios derived from the model’s coefficients. To assess the availability of habitat classes we simulated random points 35 times the number of fixes inside the individual home ranges (95% UD). The number of random points was decided by assessing the change in parameter values when increasing the number of random points from five to 100 times the number of fixes as described by Fieberg et al. (2021). Each fix and random point were assigned the value of the habitat class it landed on. We also calculated the distance to the nearest water body, house and agricultural field for each GPS fix and random point. These variables are hereafter referred to as proximity variables. We did not include the proximity to the nearest agricultural field in the southern coastal region because not every raccoon dog had agricultural fields in their home range and the closest ones were situated across the sea. Lakes and sea were omitted from the analyses because they were not used by raccoon dogs.

We made RSF models separately for all three regions and both seasons because the landscapes differ considerably in their habitat composition and differences are expected between the seasons. We also made a model for each region to compare the selection between seasons. All the models had a binary response variable with a value of 0 for random points and 1 for GPS fixes. Predictor variables used were the habitat class and the proximity variables, models comparing the seasons had the interactions of season and aforementioned variables as the variables of interest. All the models also included raccoon dog identities as a random effect. Models comparing seasons only included data from raccoon dog individuals that were followed during both study seasons (southern inland region *n* = 6, southern coastal region *n* = 6, northern region *n* = 8). The spring/summer models had respective sample sizes of 7, 7 and 10, and the autumn/winter models had sample sizes of 6, 6 and 9 for the regions.

We created all these models by assigning weights of 5000 to the random points representing availability, as suggested by Fieberg et al. (2021). According to Fieberg et al. (2021), the point of weights is to facilitate the interpretation of the model parameters. Categorical variables in logistic regression require one level to be set as the reference level which is used as a reference for the other levels. We chose the habitat class forest to be the reference level because the raccoon dog response to forests appeared quite neutral (odds ratio value very close to one). The odds ratios are calculated in relation to this level. When the odds ratio of habitat is over one, that habitat would be used more likely compared to forests if their coverage were equal. Habitats that were selected less can be interpreted as habitats that the raccoon dogs avoided, and the habitats that were selected more can be interpreted as habitats that were preferred.

All analyses were done using packages sf (v. 1.0-7, Pebesma 2018), ctmm (v. 0.6.2, Fleming and Calabrese 2022), lme4 (v. 1.1-30, Bates et al. 2015) and car (v. 3.1-2, Fox and Weisberg 2019) in R (R Core Team 2022).

## Results

### Home ranges

Home range sizes were larger in the northern region (on average 4349 ha ± 4316.5 SD) than in the southern regions (on average 397–1089 ha ± 314–1394 SD, *F*_2,18_ = 14.9, *P* < 0.001, *n* = 24; Table 2). Home range sizes were bigger during spring/summer than during autumn/winter in the southern inland region, but quite similar in the other two regions (*F*_2,17_ = 4.07, *P* = 0.036; see Table 2, *n* = 20).

**Table 2:** Mean home range sizes of raccoon dogs (95% and 50% utilization distributions) during the summer/spring season (April–July) and autumn/winter season (August–March) of waterfowl

Region	Utilization distribution	Spring/summer		Autumn/winter	
		Mean ± SD (ha)	*n*	Mean ± SD (ha)	*n*
Southern inland region	95	396.5 ± 313.9	7	113.4 ± 75.7	6
	50	73.5 ± 63.3		22.8 ± 17.0	
Southern coastal region	95	1098.0 ± 1394.0	7	1762.9 ± 1919.7	6
	50	256.5 ± 317.0		339.0 ± 373.8	
Northern region	95	4349.3 ± 4316.5	10	3883.0 ± 3671.8	9
	50	778.6 ± 702.1		642.5 ± 385.4	

### Habitat selection of raccoon dogs during spring/summer

**Fig. 3:**
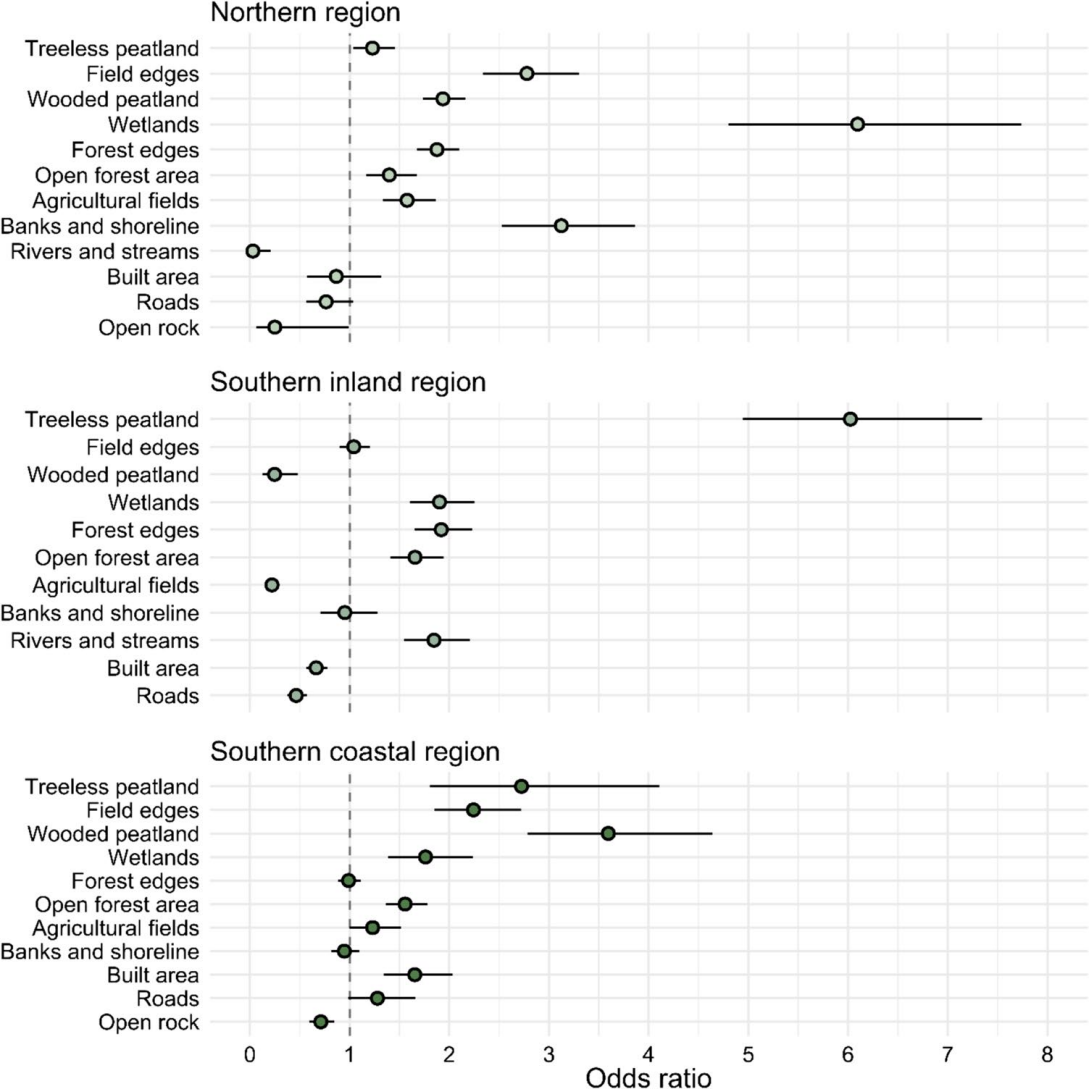
Habitat preferences in the three regions during the spring/summer season as odds ratios. The selection of every habitat is compared to the forest habitat, which is selected by raccoon dogs with no preference in any direction. Odds ratios close to zero indicate dispreference in proportion to that value. Odds ratios greater than one are preferred by the raccoon dogs in that region. Lines indicate 95% confidence intervals. The northern region *n* = 10, the southern coastal region *n* = 7, the southern inland region *n* = 7

Raccoon dogs showed clear preferences for wetlands in all three regions, and for treeless peatlands, field edges and wooded peatlands in two of the regions (Fig. 3). There was no clear universal avoidance of any other habitat than open rock areas (Fig. 3, supplementary Table 2), but they were not present in the southern inland region. It needs to be noted that in the northern region, a big part of the class “banks and shoreline” was riverbanks, so the raccoon dogs avoided open water of the rivers but strongly preferred their banks.

**Fig. 4:**
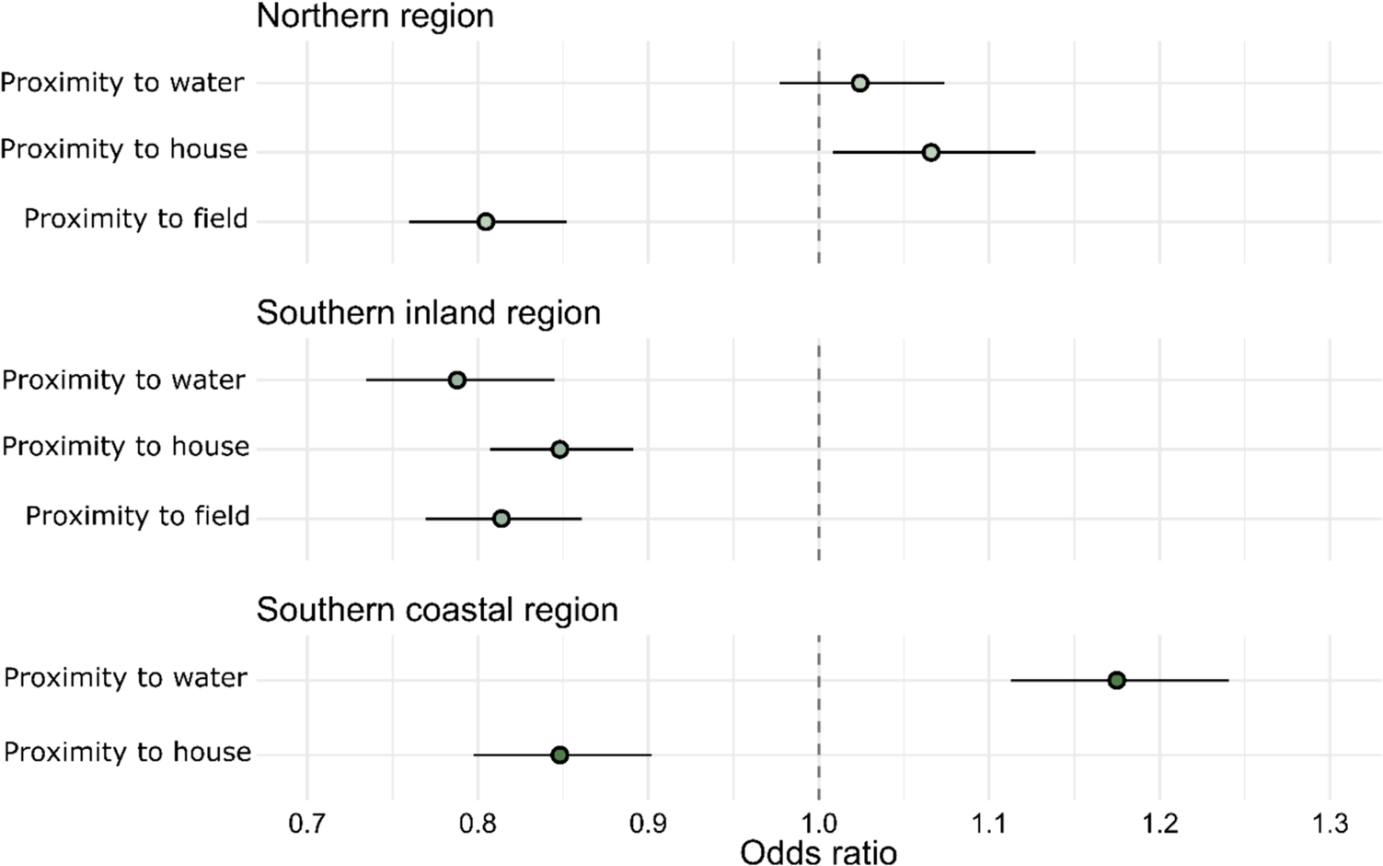
Proximity preferences during the spring/summer season as odds ratios. Values closer to zero mean preference for proximity and values equal to one (dashed line) mean no preference. Lines indicate 95% confidence intervals. The northern region *n* = 10, the southern coastal region *n* = 7, the southern inland region *n* = 7

Regarding the selection of habitat edges during spring/summer, raccoon dogs preferred field edges more than interior areas in all three regions, indicating that the use of agricultural fields is mostly focused on the edges (Fig. 3). Similarly, forest edges were preferred more than interior forest areas in the southern inland region and the northern region, but not in the southern coastal region (Fig. 3). Proximity to houses was preferred in the southern regions but not in the northern region (Fig. 4). Proximity to water was preferred in the southern inland region while longer distances to water (sea) were preferred in the southern coastal region (Fig. 4).

### Comparing habitat selection between seasons

**Fig. 5:**
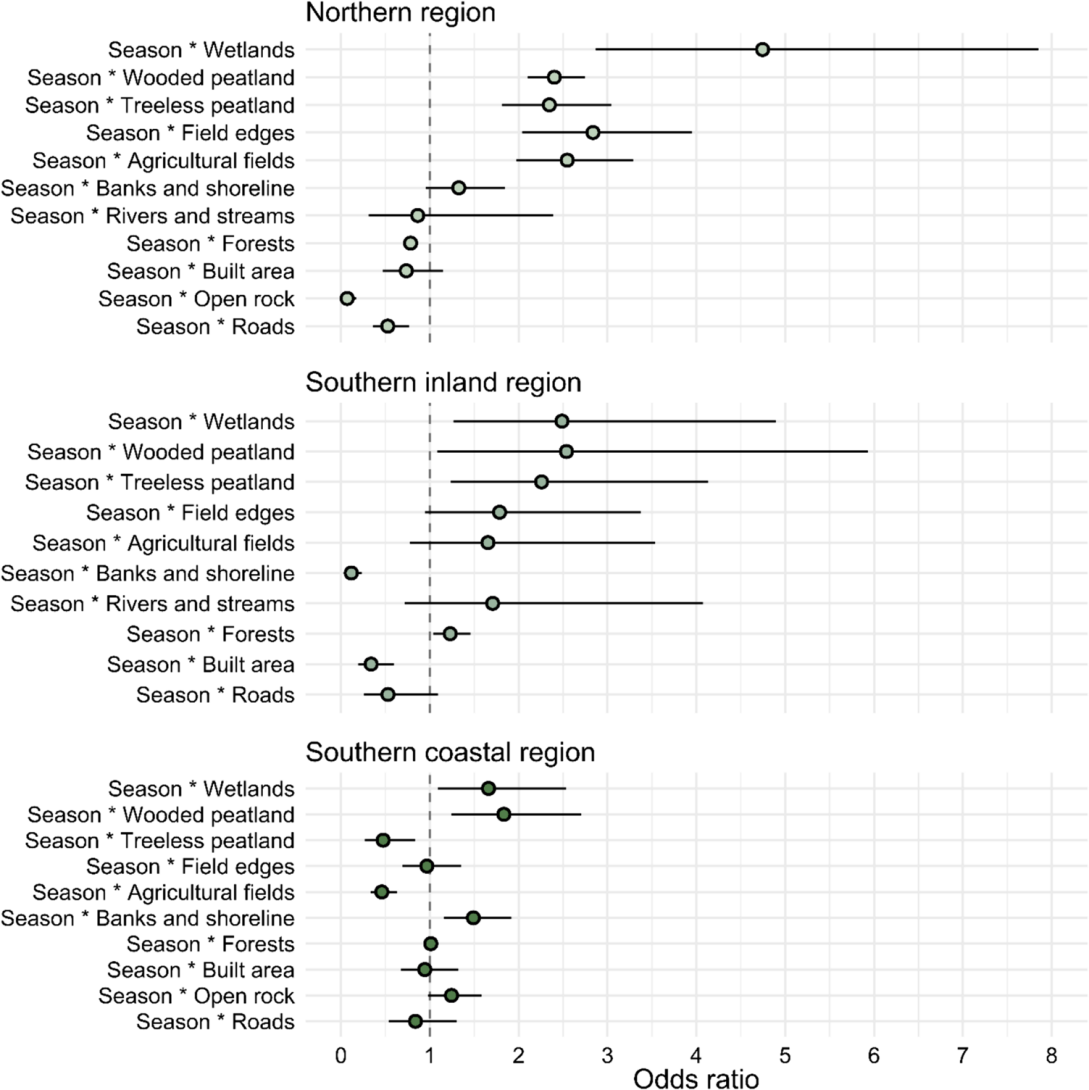
Temporal preferences between the spring/summer and the autumn/winter seasons as odds ratios. Values equal to one mean equal preference during seasons. Values above one mean stronger preference during spring/summer and values below one mean stronger preference during autumn/winter. Lines indicate 95% confidence intervals. The northern region *n* = 8, the southern coastal region *n* = 6, the southern inland region *n* = 6

During autumn/winter (outside the breeding season of ground-nesting birds and amphibians), raccoon dogs did not prefer wetlands, and treeless peatlands were avoided in the northern region (supplementary Fig. 1). When comparing the preferences between the two seasons (spring/summer vs autumn/winter), the raccoon dogs preferred wetlands and wooded peatlands more in all three regions during spring/summer than autumn/winter (Fig. 5). This was also true with treeless peatland in the southern inland and the northern regions (Fig. 5). Banks and shorelines were preferred less during spring/summer in the southern inland and the northern regions than autumn/winter, even though they were preferred more during spring/summer in the southern coastal region. Higher preference during the spring/summer can also be seen in agricultural fields and field edges in the northern region (Fig. 5). All these differences in preference were statistically significant.

## Discussion

We studied variation in home range sizes and temporal and spatial variation in habitat selection within the home ranges of GPS-collared raccoon dogs in three different landscapes. We found home range sizes to be larger in the northern region (northern and middle boreal zones) than in the southern regions (southern and hemiboreal zones). Raccoon dogs preferred wet habitats, such as wetlands and treeless peatlands in their habitat selection during spring/summer which is the breeding season of ground-nesting birds and amphibians. These preferences may lead to an elevated risk for prey species in these habitats. In addition, the raccoon dogs showed a preference for edge areas, in particular to those of agricultural fields.

### Home ranges

Home ranges were the largest in the northern region. This is in line with a somewhat common pattern seen in other mammal species where the home-range size increases with the increase of latitude and altitude associated with colder climates and a decrease in productivity (Harestad and Bunnel 1979; Damuth 1981; Dahle and Swenson 2003; Dahle et al. 2006; Rautio et al. 2013). Home ranges were also larger in the southern coastal region than in the inland region, which might be explained by a higher quantity of poor-quality habitats (open rock) and a lower quantity of preferred habitats (Table 1). However, these possible effects were not tested in the current study.

Estimates of home ranges in the northern region were clearly larger than reported in previous studies (on average 4349 ha during spring/summer) A study in northern Sweden reported a mean home range size of 1084 ha (Herfindal et al. 2016, using minimum convex polygon). In the southern regions (inland: 396 ha, coast: 1098 ha) the estimates are more similar to earlier reported home ranges (e.g. Drygala et al. 2008). Previous studies done in southern Finland (southern boreal zone) found the home ranges to be 950 ha (Kauhala et al. 1993, using the harmonic mean method) or between 90 and 261 ha (Kauhala et al. 2010, using kernel density estimation). Many of these studies used VHF-based radiotracking instead of satellite tracking (Kauhala et al. 1993, 2010; Drygala et al. 2008).

The method we used to estimate home ranges (AKDE) is known to estimate larger home ranges than kernel density estimation (KDE) or minimum convex polygon (MCP, Fleming et al. 2015). Thus, earlier home range estimates by e.g. Kauhala et al. (2010) and Herfindal et al. (2016) may at some level be underestimated due to the used methods (KDE vs MCP). Usually estimates based on VHF-tracking have been based on fewer locations (70 locations per home range, Drygala et al. 2008; Kauhala et al. 2010; Sutor and Schwarz 2012), while our estimates from the breeding season are based on an average of 376 locations per home range (see supplementary Table 1). For these reasons we believe our estimates to represent the home ranges of raccoon dogs better than previous estimates, meaning that the home ranges are most likely bigger and raccoon dogs are capable of foraging within larger areas than previously thought.

### Habitat selection of raccoon dog and implications for potential prey

The habitat preferences found in this study may lead to an elevated risk for wetland-associated prey during spring and summer. Wetlands were consistently preferred in all three regions during the breeding season of ground-nesting birds and amphibians but not outside this season. Peatlands, shorelines and riverbanks were preferred in a similar fashion in some of the regions. These habitats are important for amphibians and different nesting waterfowl and other wetland bird species, and they are also rich in insects. According to dietary studies of raccoon dogs living close to waterbodies, amphibians and reptiles are most commonly found in their diet, at least in spring (Tuomikoski et al. 2024). As outlined in the introduction the effect of raccoon dogs on breeding bird populations is less clear. In some study systems, there is an indication that raccoon dog removal may have positive impacts on the breeding success of waterbirds (Nummi et al. 2019; Jaatinen et al. 2022). However, Jaatinen et al. (2022) did not differentiate the effect of raccoon dog from the effect of American mink (Jaatinen et al. 2022) and Nummi et al. (2019) did not find significant results for single species instead of communities. On the contrary, Kauhala (2004) did not found the raccoon dog to be important at all to the breeding success of birds. According to various artificial nest predation studies, raccoon dogs increase the nest-predation rate on shorelines (Dahl and Åhlén 2018; Holopainen et al. 2020, 2021), forests (70–1400 m from the shoreline) and wetland habitats (Holopainen et al. 2020, 2021), and the raccoon dog is the most common mammalian predator of these habitats (Holopainen et al. 2021; Selonen et al. 2022b). However, artificial nest predation studies may not capture the true nest predation well enough (Moore and Robinson 2004). This might explain why the predation on nests is not seen clearly in dietary studies (Tuomikoski et al. 2024). In any case, the preference for wetland habitats, regardless of the driver, might lead the opportunistic raccoon dog to the nests of water birds more than would be expected without the preference. Certainly, the observed habitat preferences of raccoon dog in general increase the predation risks of wetland-associated prey species. It is also notable, that currently this invasive mesopredator is the most common mesopredator in southern Finland, being clearly more abundant near wetlands than the native mesopredators, the red fox and the badger (Selonen et al. 2024a, b).

Despite the above preferences, the raccoon dogs are mostly generalists in their use of habitats, meaning that practically no habitat is left unused. This was most pronounced in the coastal region where raccoon dogs did not avoid almost any habitat. True to the generalist nature of the species, most used habitats were also the most common habitats of the landscape, which means that forests were most utilized while not preferred or avoided by raccoon dogs. In earlier studies, the selection of forests varies, but so do the methods to classify them. Coniferous forests were preferred in a German study (Sutor and Schwarz 2013) or neutral in Danish study (Wooldridge et al. 2024) and avoided in Northern and Eastern European studies (Kauhala and Auttila 2010; Melis et al. 2015) while deciduous forests were avoided (Sutor and Schwarz 2013) or preferred (Wooldridge et al. 2024) and mixed forests preferred (Kauhala and Auttila 2010). Agricultural fields of the southern inland region were abundant in the landscape but heavily avoided by the raccoon dog, which is also in line with earlier studies showing avoidance or the least preference for agricultural fields (Drygala et al. 2007; Kauhala and Auttila 2010; Sutor and Schwarz 2013; Wooldridge et al. 2024).

One source of variation in our study is the limitations in classifying habitat classes. The definition of some habitat classes may vary between regions. For example, wooded peatland is a particularly difficult class to classify because of the extensive ditching of peatlands in Finland (Vasander et al. 2003). Many of the areas classified into this class are peatlands turned into commercial forests. The proportion of wooded peatland, ditched or not, is also higher in the northern boreal and middle zones than in the southern and hemiboreal zones. This might explain some of the variation in results between the regional models. Another class inhibiting lots of variation between the models is the class of treeless peatlands. This might be because of a high proportion of treeless peatlands in the northern region and the fact that in the study areas of the southern inland region, treeless peatlands were overgrown lakes or ponds. Treeless peatland was highly preferred in regions where the class consisted more of these overgrown lakes and ponds than conventional mires.

Our study also indicates edge selection for raccoon dogs. This aspect has been left out in previous raccoon dog habitat selection studies, but habitat edges are important for foraging and movement patterns for other mammalian predators, including red foxes (e.g. Bischof et al. 2018; Šálek et al. 2010, 2014; Sonerud 2022). Preference for habitat edges (or “habitat margins”) is of concern for prey species that use these areas. For example, field margins are preferred by pheasants (*Phasianus colchicus*) and partridges (*Perdix perdix*) for brood-rearing (Krüger et al. 2023). Raccoon dogs are able to eat eggs and chicks, so if these are located on the field margins along with other food sources, raccoon dogs might be a threat to them. Nesting near forest edges is sometimes found to increase the nest predation risk (Manolis et al. 2002), but usually this is not the case in studies of edge effects on nest predation (Hanski et al. 1996; Lahti 2001). However, according to an artificial nest predation study by Krüger et al. (2018), mammalian predation (including raccoon dogs) was on average more active close to the forest-field edge on both sides while avian predation was more intense further from the edge. Nests over 20 m from the edge suffered high predation by raccoon dogs (Krüger et al. 2018). Raccoon dogs actively use both forest interior and edges but prefer edges, which may increase the predation risk at the edges, usually between forests and fields.

Our findings regarding wetlands and other wet habitats are supported by earlier studies. Studies that included those habitats also showed preferences for them by raccoon dogs (Melis et al. 2015; Schwemmer et al. 2021; Wooldridge et al. 2024). To our knowledge, only one other study included peatland habitats. Wooldridge et al. (2024) very recently found raccoon dogs to prefer peatbogs (peatlands) and marshes (wetlands) throughout the year in Denmark. Further studies are needed to investigate the possible causal nature and consequences of these correlations in the field.

## Conclusions

Our study shows that the invasive raccoon dog prefers and spends time in the same wetland and peatland habitats that several endangered ground-nesting birds use for nesting during spring and summer. This way the invasive raccoon dog may pose a risk for nesting of endangered wetland-associated species. Wet habitats, such as treeless peatlands and wetlands are abundant in several other food sources than birds, such as insects and amphibians which are regularly eaten by raccoon dogs (Mulder 2012; Tuomikoski et al. 2024). It is not clear what drives raccoon dogs to these habitats, but these spatiotemporal correlations may at least partially explain the high predation risk observed in artificial nest predation studies near wetlands (e.g. Holopainen et al. 2020, 2021). The risk the raccoon poses for wetland-associated species is further emphasized by the potentially high numbers of this invasive species near wetlands in southern Finland (Selonen et al. 2024a, b).

## Supplementary Information

Below is the link to the electronic supplementary material.

Supplementary file1 (DOCX 9216 KB)

## Data Availability

The movement data of the animals is produced and therefore owned by the Finnish Wildlife Agency and the Ministry of Agriculture and Forestry, but the authors have permission to use it and publish it in the form of scientific articles. All the datasets are available from the corresponding author on a reasonable request.

## Code availability

Used code is available from the corresponding author on a reasonable request.
