# Supplementary material for: The habitat preferences of invasive raccoon dog imply elevated risks for wetland-associated prey species
Source: Oecologia. 2024 Sep 4;206(1-2):73–85. doi: 10.1007/s00442-024-05614-y (PMC11489220; doi:10.1007/s00442-024-05614-y)
Supplement: Supplementary file 1 — Supplementary file1 (DOCX 9216 KB) [file 442_2024_5614_MOESM1_ESM.docx]

The habitat preferences of invasive raccoon dog imply elevated risks for wetland-associated prey species

Toivonen, Pyry^1*^, Laaksonen, Toni^1^, Piironen, Antti^1^ & Selonen, Vesa^1^

1 Department of Biology, University of Turku, FI-20014 Turku, Finland

* pntoiv@utu.fi

***Supplementary information***

**Table 1.** Basic information about the raccoon dogs used in the study. Animal ID is the unique ID assigned to the individual in different regions (HB coast = the southern coastal region, SB inland = the southern inland region and N-MB = the northern region). The tracking start date is the date of the first GPS fix and the tracking end date is the date of the last GPS fix. Number of fixes is the total number of GPS fixes from the individual used in the analyses (in parentheses the number of fixes during the breeding season of ground-nesting birds). Partner removals is the number of partners that were removed from the individual (by hunters) during the tracking period.

| **Animal ID** | **Sex** | **Region** | **Tracking start date** | **Tracking end**  **date** | **Number of fixes (spring/summer)** | **Partner removals** |
| --- | --- | --- | --- | --- | --- | --- |
| 103 | Male | HB coast | 28.9.2020 | 15.7.2021 | 862 (383) | 2 |
| 105 | Male | HB coast | 12.2.2020 | 12.11.2021 | 1042 (369) | 1 |
| 107 | Male | HB coast | 29.10.2020 | 30.12.2021 | 1223 (460) | 3 |
| 108 | Female | HB coast | 14.2.2020 | 19.8.2020 | 710 (452) | 0 |
| 109 | Female | HB coast | 30.8.2019 | 28.5.2020 | 948 (163) | 0 |
| 110 | Male | HB coast | 9.1.2021 | 7.10.2021 | 1275 (452) | 1 |
| 111 | Male | HB coast | 5.4.2022 | 15.7.2022 | 355 (355) | 1 |
| 202 | Female | SB inland | 13.3.2022 | 12.4.2022 | 290 (96) | 0 |
| 203 | Female | SB inland | 14.1.2022 | 14.4.2022 | 363 (163) | 0 |
| 205 | Female | SB inland | 20.2.2022 | 28.6.2022 | 635 (607) | 0 |
| 206 | Female | SB inland | 17.3.2022 | 28.6.2022 | 1038 (960) | 0 |
| 207 | Female | SB inland | 19.3.2021 | 9.6.2021 | 448 (420) | 0 |
| 208 | Male | SB inland | 12.3.2021 | 18.5.2021 | 176 (138) | 0 |
| 211 | Female | SB inland | 29.4.2021 | 13.6.2021 | 447 (447) | 0 |
| 301 | Female | N-MB | 24.5.2017 | 12.10.2017 | 197 (0) | *NA* |
| 303 | Female | N-MB | 23.5.2017 | 8.11.2017 | 574 (197) | *NA* |
| 304 | Male | N-MB | 27.10.2017 | 7.8.2018 | 935 (490) | 1 |
| 306 | Male | N-MB | 28.9.2017 | 21.8.2019 | 2239 (651) | 2 |
| 307 | Female | N-MB | 12.5.2019 | 7.10.2019 | 554 (309) | 0 |
| 308 | Male | N-MB | 20.5.2020 | 14.9.2020 | 404 (240) | 0 |
| 309 | Male | N-MB | 20.2.2020 | 4.8.2020 | 111 (111) | 1 |
| 310 | Female | N-MB | 4.6.2021 | 15.10.2021 | 526 (225) | 1 |
| 311 | Male | N-MB | 18.6.2021 | 16.10.2021 | 476 (172) | 0 |
| 312 | Male | N-MB | 14.12.2021 | 16.8.2022 | 414 (294) | 0 |
| 313 | Male | N-MB | 5.10.2021 | 13.8.2022 | 251 (251) | 1 |

**Table 2.** The results of logistic regression models for the three regions during spring and summer months. The variable column includes the habitat classes. Intercept contains the reference level which is the forest class. Asterisks denote the statistical significance of the coefficient. Odds ratios were conducted by exponentiating the coefficients shown in the table.

| **Variable** | **Southern inland region** | **Southern coastal region** | **Northern region** |
| --- | --- | --- | --- |
|  | **Estimate (± SE**) | **Estimate (± SE)** | **Estimate (± SE)** |
| Intercept | -12.059* (± 0.105) | -11.717* (± 0.284) | -12.439* (± 0.062) |
| Built area | -0.409* (± 0.080) | 0.514* (± 0.106) | -0.143 (± 0.213) |
| Roads | -0.767* (± 0.107) | 0.254 (± 0.133) | -0.269 (± 0.154) |
| Agricultural fields | -1.509* (± 0.082) | 0.215* (± 0.106) | 0.455* (± 0.086) |
| Field edges | 0.041 (± 0.074) | 0.815* (± 0.099) | 1.022* (± 0.087) |
| Forest edges | 0.652* (± 0.077) | -0.013* (± 0.059) | 0.629* (± 0.057) |
| Open forest area | 0.505* (± 0.081) | 0.440* (± 0.068) | 0.335* (± 0.092) |
| Banks and shoreline | -0.049 (± 0.152) | -0.063 (± 0.076) | 1.139* (± 0.108) |
| Wooded peatland | -1.399* (± 0.338) | 1.279* (± 0.130) | 0.661* (± 0.056) |
| Treeless peatland | 1.800* (± 0.101) | 1.002* (± 0.210) | 0.206 (± 0.087) |
| Rivers and streams | 0.613* (± 0.090) | *NA* | -3.503* (± 0.981) |
| Wetlands | 0.643* (± 0.087) | 0.570* (± 0.123) | 1.808* (± 0.122) |
| Open rock | *NA* | -0.353* (± 0.090) | -1.388* (± 0.704) |
| Proximity to field | -0.206* (± 0.029) | *NA* | -0.217* (± 0.029) |
| Proximity to water | -0.238* (± 0.036) | 0.172* (± 0.029) | 0.024 (± 0.024) |
| Proximity to house | -0.165* (± 0.025) | -0.139* (± 0.028) | 0.064* (± 0.028) |


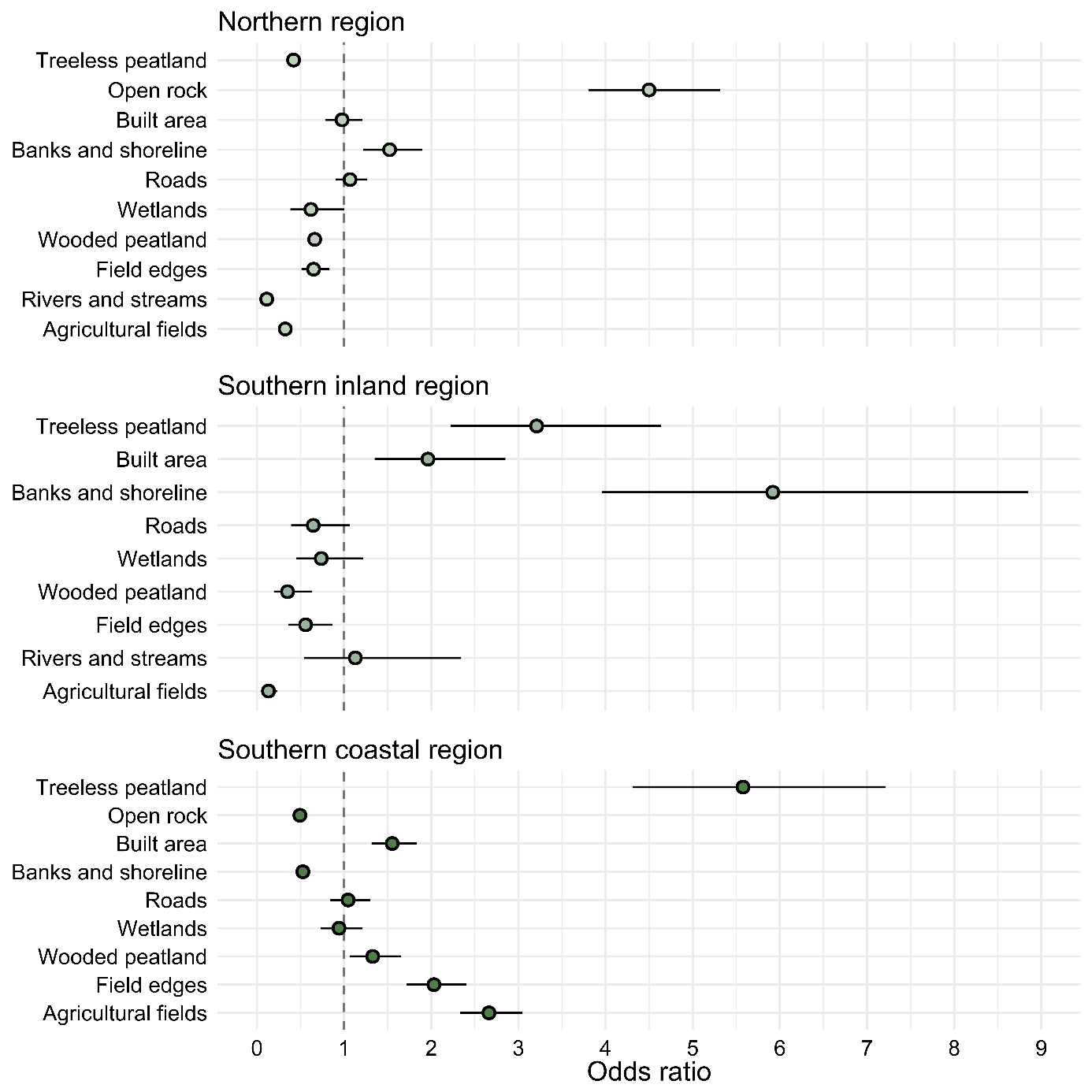


**Figure 1.** Habitat preferences during autumn and winter months as odds ratios. The selection of every habitat is compared to the forest class, which is selected by raccoon dogs with no preference in any direction. Odds ratios close to zero indicate dispreference in proportion to that value. Odds ratios greater than one are preferred by the raccoon dogs in that region. Lines indicate 95 % confidence intervals. The northern region n = 9, the southern coastal region n = 6, the southern inland region n = 6.


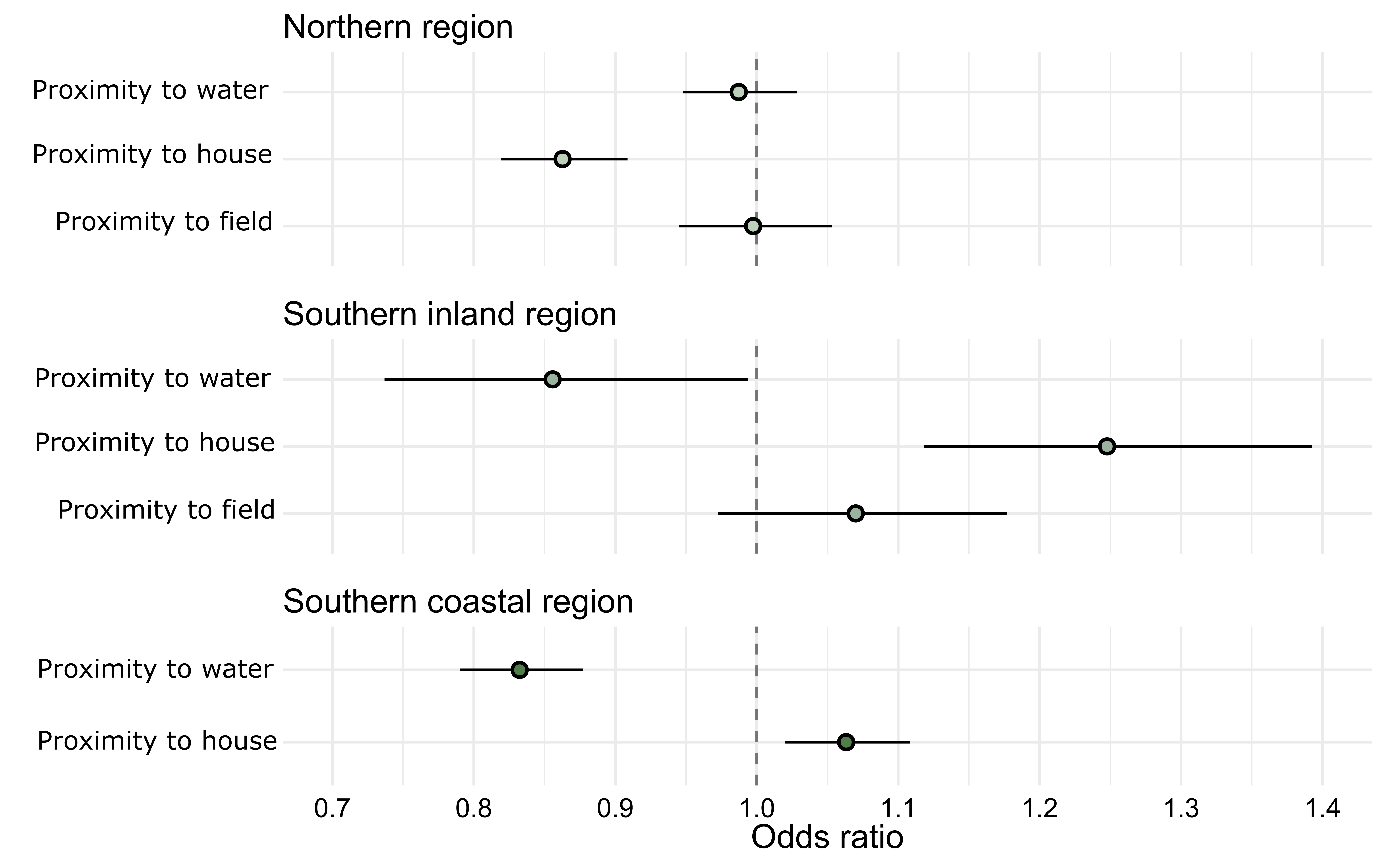


**Figure 2.** Proximity preferences during autumn and winter months as odds ratios. Values closer to zero mean preference for proximity and values equal to one (dashed line) mean no preference. Lines indicate 95 % confidence intervals. The northern region n = 9, the southern coastal region n = 6, the southern inland region n = 6.

*
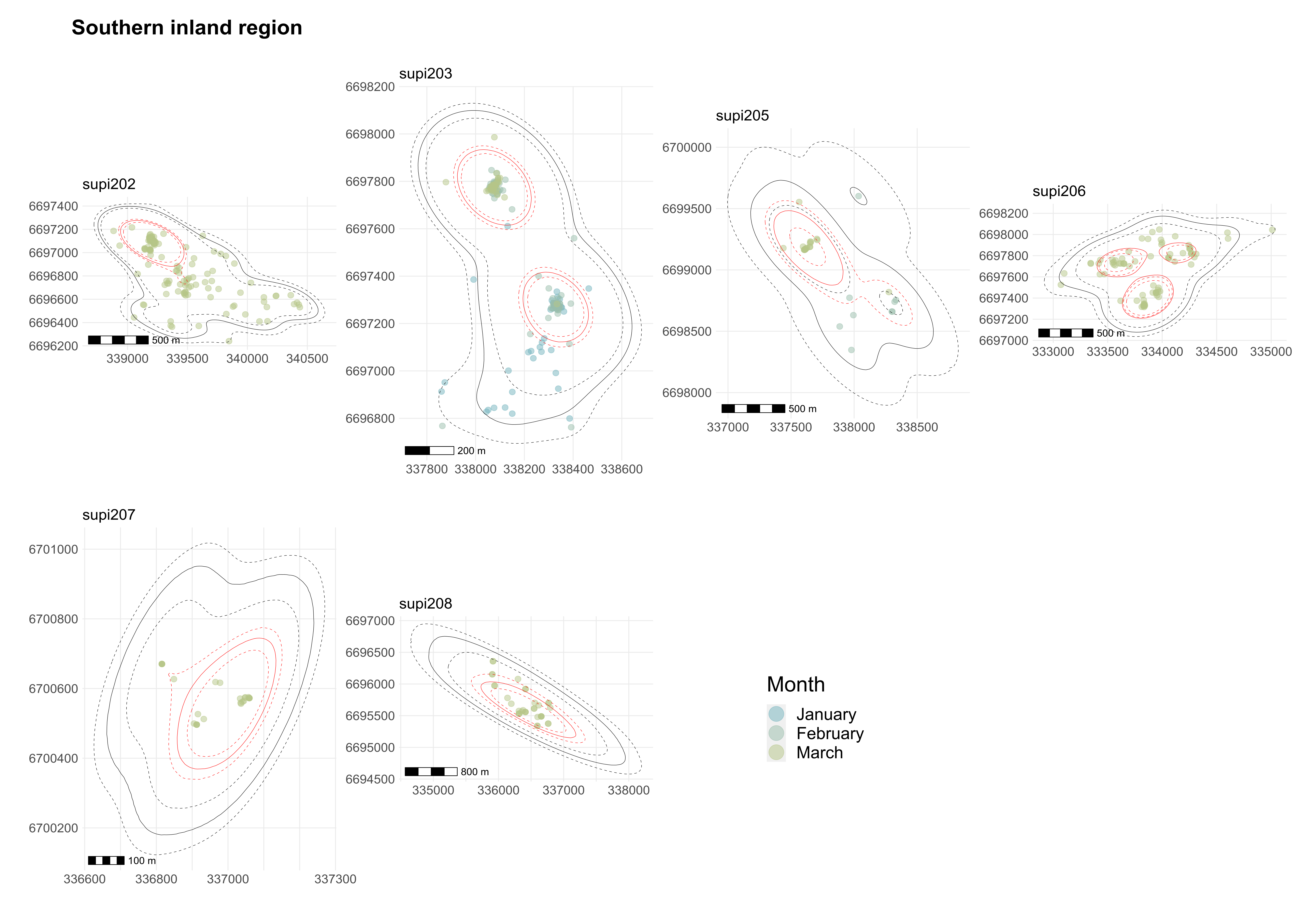
*

**Figure 3.** Home ranges and GPS-fixes from autumn and winter months in the southern inland region. Months are visualized in different colors. Solid black lines are the estimated home ranges and dashed lines their 95 % confidence intervals. Home ranges and fixes shown within the solid lines were used in the analyses.


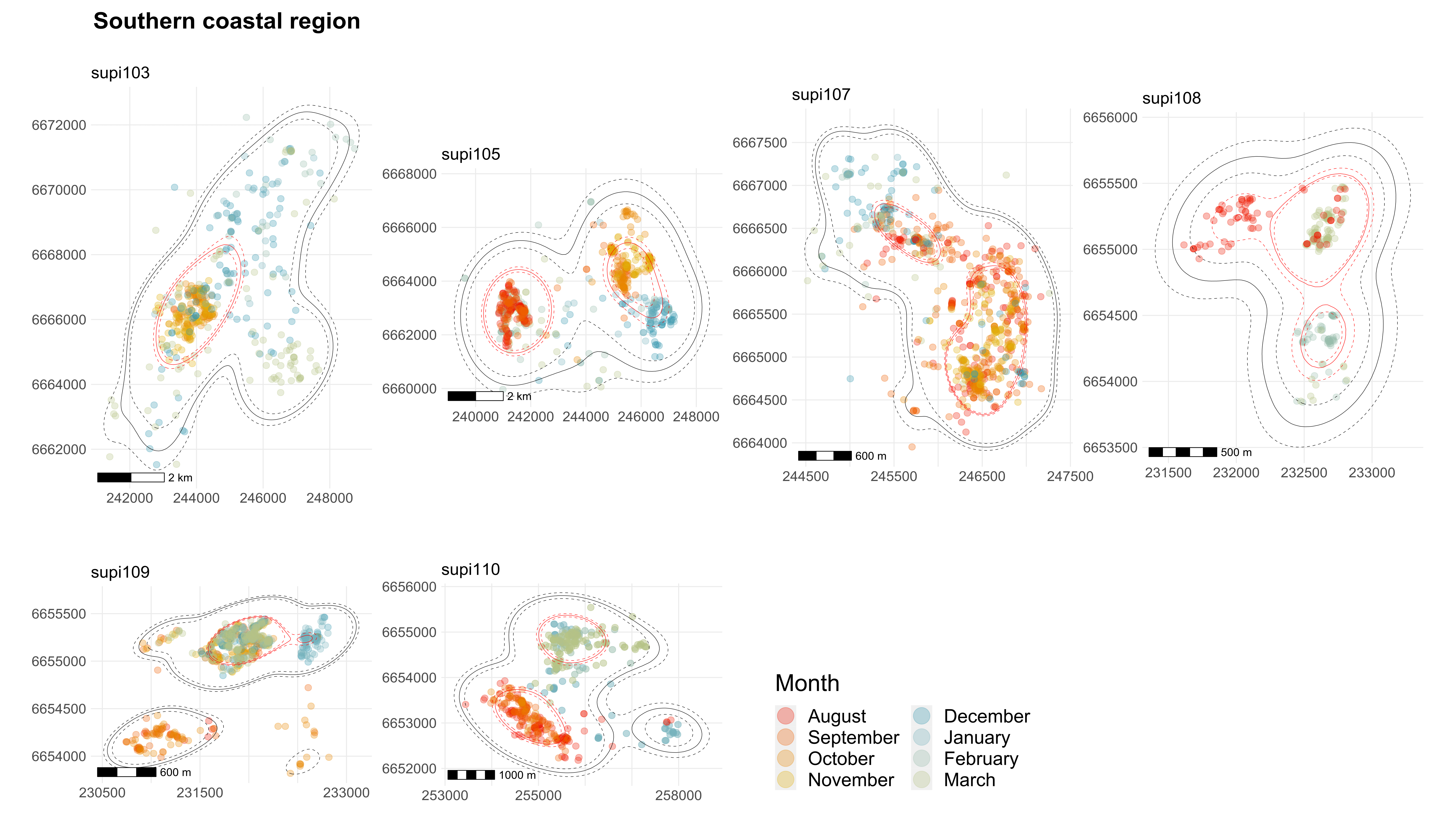


**Figure 4.** Home ranges and GPS-fixes from autumn and winter months in the southern coastal region. Months are visualized in different colors. Solid black lines are the estimated home ranges and dashed lines their 95 % confidence intervals. Home ranges and fixes shown within the solid lines were used in the analyses.


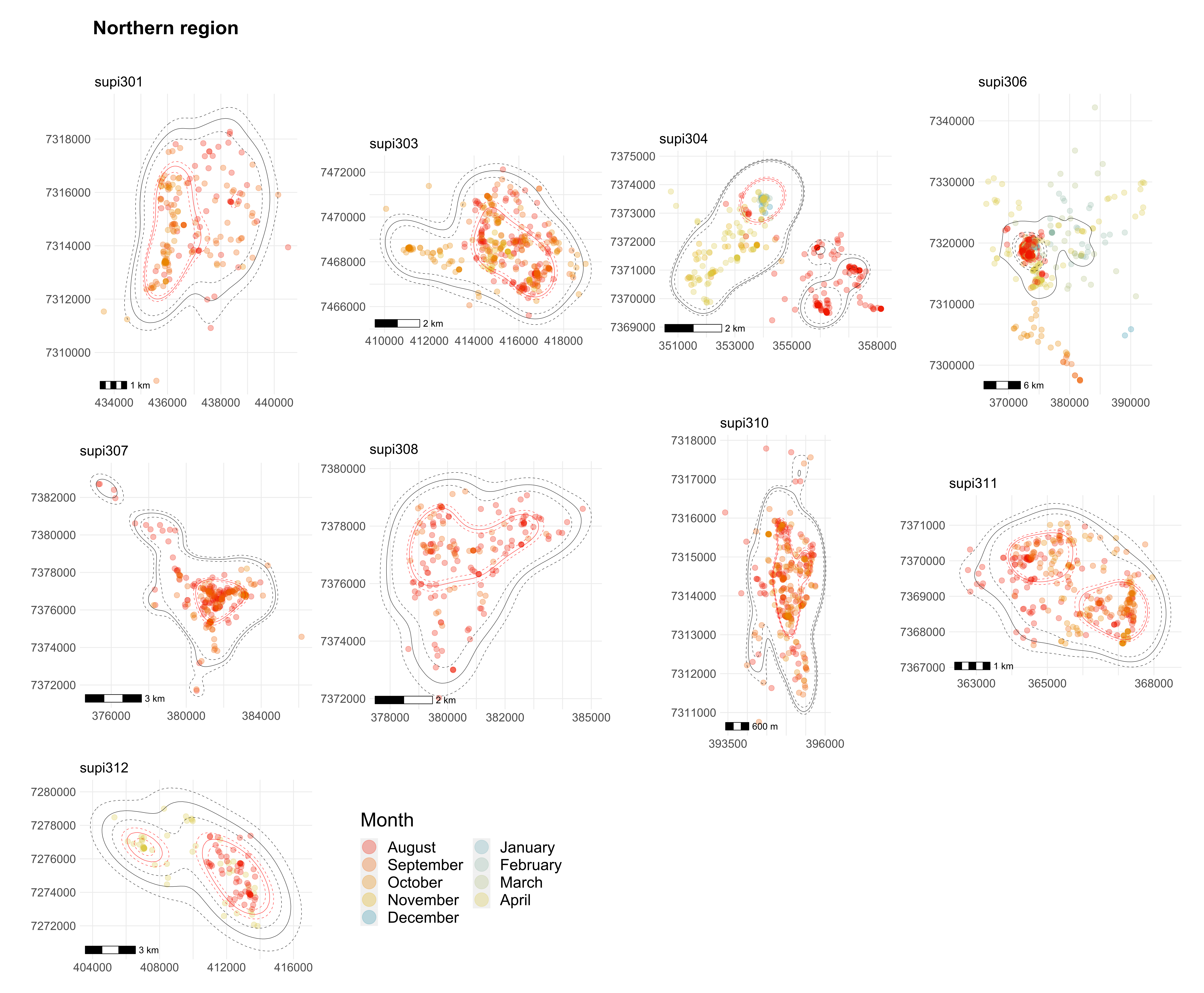


**Figure 5.** Home ranges and GPS-fixes from autumn and winter months in the northern region. Months are visualized in different colors. Solid black lines are the estimated home ranges and dashed lines their 95 % confidence intervals. Home ranges and fixes shown within the solid lines were used in the analyses.
